# Managing parental capacity concerns in IVF: the role of child welfare committees—a survey of unit directors

**DOI:** 10.1007/s10815-025-03529-y

**Published:** 2025-06-02

**Authors:** Avi Tsafrir, Tamar Artom, Raoul Orvieto, Jordana H. Hyman, Zivit Worcman, Shlomit Tsafrir

**Affiliations:** 1 Faculty of Medicine, Hebrew University of Jerusalem, Jerusalem, Israel; 2 Department of Obstetrics and Gynecology, IVF Unit, Shaare Zedek Medical Center, Jerusalem, Israel; 3 Fertility Association Board, Tel Aviv, Israel; 4 Israel Fertility Association Psychological Counseling in Fertility Care Group, Tel Aviv, Israel; 5 Infertility and IVF Institute, Sheba Medical Center, Ramat Gan, Israel; 6 Faculty of Medical and Health Science, Tel Aviv University, Tel Aviv, Israel; 7 Center for Sexual Health, Sheba Sheba Medical Center, Ramat Gan, Israel; 8 The Child and Adolescent Psychiatry Division, Sheba Medical Center, Ramat Gan, Israel; 9 Faculty of Medical & Health Sciences, Tel Aviv University, Tel Aviv, Israel

**Keywords:** Fertility treatments, Parental capacity, Parental capability, Child welfare, IVF, Assisted reproductive technology

## Abstract

**Purpose:**

To examine whether Child Welfare Advisory Committees (CWCs) assist IVF unit directors in addressing concerns about prospective parental capacity (PC).

**Methods:**

An anonymous online survey of 26 IVF unit directors in Israel was conducted in November 2023, focusing on referrals to CWCs, reasons for referral, and satisfaction with CWC services.

**Results:**

Of the 26 directors, 23 responded (88%). Over a 3-year period, 21 (91%) reported referring candidates to CWCs, with referral counts ranging from 1 to “over 30.” The most frequently cited concerns were cognitive developmental disability (16 directors, 72%), mental illness (also 16, 72%), and severe physical disability (10, 43%). Thirteen directors (57%) rated CWC availability and quality as reasonable, while five (21%) found services insufficient, and three (13%) reported no access at all. Ten directors (43%) used alternative strategies, including denying treatment without CWC input, referring patients elsewhere, or prolonging the assessment to encourage withdrawal. One in four respondents reported facing legal or administrative challenges following further evaluation requests or treatment refusals.

**Conclusions:**

Concerns about prospective PC are relatively uncommon but present in all IVF units. The most frequent concerns relate to cognitive developmental disability, mental illness, and severe physical disabilities. While most directors consult CWCs in such cases, satisfaction with their function is mixed. These findings highlight the need for clearer guidelines and more accessible, consistent consultation structures to support clinicians in managing complex cases.

**Supplementary Information:**

The online version contains supplementary material available at 10.1007/s10815-025-03529-y.

## Introduction

Child welfare risks represent a serious global concern. In 2021, the U.S. reported that 8.1 out of 1000 children were victims of abuse or neglect [1]. In the UK, 4.3 out of 1000 children were on child protection plans [2]. Parental risk factors—such as substance abuse, mental health disorders, single parenthood, or incarceration—are identifiable before pregnancy [3]. This raises ethical dilemmas for fertility clinicians and counselors regarding the prospective parental capacity of patients seeking fertility treatment, including whether to deny treatment based on potential future risks to the child.

The “welfare of the child” principle in reproductive medicine emphasizes the clinician’s ethical obligation to consider the well-being of the future child. However, the extent of clinicians’ responsibility to assess PC and possibly deny treatment remains a subject of debate. A full presentation of the philosophical and bioethical aspects of this issue is beyond the scope of this text and has been discussed extensively elsewhere [4–13]. It is suggested that individuals with infertility have the same reproductive rights as those who are fertile. As fertile individuals do not require approval to reproduce, the same standard should apply to those requiring medical assistance. Therefore, withholding fertility treatment on the basis of concerns regarding PC constitutes discrimination against infertile individuals. While child welfare assessments aim to protect future children, they may unintentionally contribute to reproductive stratification. For example, in Sweden, assessments are mandated for ART using donated gametes, which can disproportionately impact non-heteronormative families [14]. Such disparities highlight the need for transparent, equitable assessment criteria. On the other hand, unlike spontaneous reproduction, clinicians are involved in the reproductive process of infertile patients, and *carry joint responsibility for the welfare of the child because of his or her causal and intentional contribution to the parental project* [4]. Hence, the clinicians have a moral duty to refuse collaboration if there is a high risk of serious harm to the future child, as stated by the ESHRE taskforce: *The fertility specialist should refuse to collaborate in the parental project of the would-be parents if he or she judges that there is a high risk of serious harm to the future child* [4]. Similarly, the American Society for Reproductive Medicine Ethics Committee permits the withholding of services when there is a well-founded belief that prospective parents may not provide adequate care [5].

This predicament is not merely theoretical, but also a practical concern for fertility clinicians and counselors, leading to many questions: who is entitled or obligated to refer infertility patients for PC assessment? Which patients should be assessed? Who makes the final decision on authorizing or denying treatment? And finally, how should clinicians communicate these decisions to patients? Fertility specialists are often reluctant to assume this judicial role, as it conflicts with their primary mission to help individuals achieve parenthood.

To address these challenging issues, countries have established different legal and professional frameworks.

In the UK, the Human Fertilisation and Embryology Authority (HFEA) code of practice, which stems from the Human Fertilisation and Embryology Act 1990, considers this issue. According to the code, clinic staff have an option to deny treatment, but are also obligated to do so in cases at risk: “The centre should refuse treatment if it: (a) concludes that any child who may be born or any existing child of the family is likely to be at risk of significant harm or neglect, or (b) cannot obtain enough information to conclude that there is no significant risk” [15]. The HFEA mandates a welfare of the child assessment for all IVF patients. This may involve completing a Welfare of the Child form [16], gathering information on the medical, psychological, and social history of prospective parents. The assessment aims to identify serious concerns about the child’s future well-being, focusing on risk factors such as substance abuse, criminal history, and the home environment. The responsibility for conducting assessments lies primarily with the clinical team within fertility clinics. While the HFEA encourages the use of clinical ethics committees for complex cases, their establishment and consultation are not mandatory. Decisions regarding treatment approval or denial are typically made by the clinic director, who may seek guidance from social services or external experts when needed.

In Norway, as summarized by Egeland et al. [17], the assessment of future PC for IVF patients is mandated by law, which requires a medical and psychosocial evaluation of the woman and any partner, taking into account their capacity for care and the child’s best interests. The evaluation must be conducted by the ART physician, who has the discretion to involve a psychologist or counselor but is not required to do so. Since 2020, a police certificate is also required, which includes a check for criminal offenses relevant to the child welfare, including abuse, physical violence, and serious drug-related crimes. However, the final decision to approve or deny treatment lies with the ART physician. Physicians in public clinics have access to clinical ethics committees for guidance, but consultation is voluntary and non-binding. If treatment is denied, patients can appeal to the County Governor or seek treatment at another clinic without the obligation to disclose the previous assessment.

According to Swedish legislation, an ART treatment with donor gametes should be offered only if it can be assumed that the child “will grow up in good circumstances.” However, when a couple’s own gametes are used, there is no legal requirement for such an assessment [7].

In the Netherlands, the assessment of future PC for IVF patients is guided by the Model Protocol on Possible Moral Contraindications in Fertility Treatments developed by the Dutch Society for Obstetrics and Gynecology [18]. The decision to provide or refuse fertility treatment is made through a multidisciplinary consultation involving physicians, psychologists, social workers, and potentially ethicists and legal advisors. Dutch law allows clinicians to refuse treatment if there is a significant concern about the child’s welfare. Physicians are not required to actively investigate reasons to deny treatment but must act if clear concerns arise. The clinic director is responsible for the final decision, though it is made within the context of the multidisciplinary team’s recommendations.

According to the Ethics Committee of the American Society for Reproductive Medicine [5] fertility programs may withhold services from prospective patients if there are well-grounded reasons to believe they will be unable to provide minimally adequate or safe care for offspring. The ASRM recommends that fertility programs develop written procedures for assessing parental capacity when concerns arise. A program’s assessment of a patient’s inability to care for a child or potential to cause harm to a child should be made jointly among members of the program and, if indicated, consultation with appropriate other professionals, and should be documented [5].

In Israel, the assessment of PC is typically court-ordered and focuses on existing children, with no clear legal framework addressing prospective parenthood before a child is born [19]. According to the recommendations of the Israeli Ministry of Health, every IVF unit in Israel must have a social worker or psychologist, and all patients are to be offered an introductory meeting during the unit’s intake process. Additionally, a psychosocial assessment may be initiated by the medical team if concerns arise regarding the patient’s suitability for treatment. In routine ART practice, fertility patients are not typically required to disclose criminal records, nor are they routinely referred for psychological evaluation unless a specific concern emerges. However, a 1990 directive issued by the Director of the Ministry of Health stipulates that if concerns arise regarding the prospective parents’ PC, a mental health professional must assess them. Based on this assessment, treatment may be denied if deemed necessary [20]. In 2012, a governmental committee established by the Ministry of Health to examine regulatory issues regarding assisted reproduction recommended the establishment of an advisory CWC in every fertility unit to alleviate the burden on individual clinicians. The advisory committee should include a gynecologist, social worker, psychologist, and a medical specialist who is not a gynecologist. Fertility clinicians may, at their discretion, refer patients to the CWC for assessment, seeking recommendations on the provision of fertility treatments [21]. The CWC would be able to share the significant onus of these decisions beyond the individual clinician’s responsibility. However, these recommendations were not fully adopted or incorporated into Israeli regulations or law, resulting in ambiguity regarding denial of fertility treatments based on questionable PC.

In certain countries, denying fertility treatments on the basis of future PC may depend on whether fertility treatments are provided in the public or private sectors [5, 7]. The Israeli healthcare system provides comprehensive medical services to all citizens, including fertility treatments. Medical care is delivered through four Health Maintenance Organizations, each responsible for providing a standardized healthcare services package. Fertility specialists may face significant pressure to provide treatment despite ethical reservations, given the perception that fertility care is a fundamental right, due to the extensive public funding for fertility care. This further complicates the medical team’s decisions regarding fertility treatment in cases of concern for PC.

The information presented above suggests that while the primary responsibility for deciding whether to provide or withhold fertility treatment in cases of PC concerns rests with the medical team, there is often a recommendation or expectation for multidisciplinary consultation, through an ethical advisory committee. However, data on the actual utilization of such committees by clinicians and the outcomes of these consultations are limited [17, 22].

To address this research gap, we conducted a comprehensive survey of all IVF unit directors in Israel, examining cases that required PC evaluations. Our study focused on the experience and satisfaction of directors with CWCs as advisory bodies.

## Methods

An anonymous online survey was distributed in November 2023 to directors of all 26 IVF units in Israel. Directors were invited to participate through a focused WhatsApp group for IVF unit directors, with the invitation issued by the president of the Israeli Fertility Association. Follow-up reminders were sent over 2 weeks, after which data collection was closed. The survey comprised eight multiple-choice questions, with an option for additional comments. Respondents were asked to share their views and experiences regarding IVF patients for whom concerns had been raised about future PC, especially regarding their interactions with CWCs in the past three years.

The full survey is provided in the supplementary material.

Given the small number of IVF units in Israel and the close-knit nature of the professional community, ensuring respondent confidentiality was crucial. To maintain anonymity, no identifying data was collected, such as personal information, patient volume and demography, or geographical location.

An institutional review board was not required, as the survey focused on clinical approach among professionals and did not involve patient details.

## Results

In this survey, 23 out of 26 IVF unit directors responded, yielding a response rate of 88%. When asked, “In the past three years, approximately how many times has your unit needed to discuss treatment for couples or women where concerns arose regarding the future child’s welfare?”, 21 respondents (93%) reported having such discussions, while one did not respond. The average number of cases discussed was 5.1 ± 6.3 (range: 0 to “over 30” (counted as 30 for analysis).

Regarding referrals to CWCs, 21 directors (91.3%) reported referring patients for assessment in the past 3 years, while one did not respond. The number of referrals per unit ranged from 1 to “over 30,” as noted by one respondent. The average number of patients referred for assessment was 3.7 ± 3.8, with considerable variation. Specifically, 12 directors (52%) reported referring 1–4 patients, five directors (21%) referred 5–9 patients, and four directors (17%) referred 10 or more patients. The most frequently cited reasons for concerns regarding PC were cognitive developmental disability (reported by 16 directors, 72%) and mental illness that may affect parental capacity (16 directors, 72%). Respondents could select more than one reason for referral. Additional reasons for referral are detailed in Table 1.

**Table 1:** Prevalence of reported indications for child welfare committee assessment and referral: 3-year nationwide survey of 23 IVF Unit Directors

Indication for concern	Number of respondents (% of all respondents)
Cognitive developmental disability	16 (70)
Mental illness that may affect parental capacity	16 (70)
Severe physical disability	10 (43)
Drug/alcohol use to an extent that may impair parental capacity	4 (17)
Criminal aspects	4 (17)
Other	2 (9)

Half of the IVF unit directors (13, 57%) rated the availability and quality of CWC services in their facilities as “reasonable.” However, five directors (21%) described the local CWC as insufficient and three (13%) reported that no CWC services were available. One director did not respond; another stated that a CWC was not needed in their unit. When asked, if the CWC service is insufficient, what do you believe is the reason? Five of eight directors suggested that the committee lacked legal authority to enforce its decisions.

Further, we asked if “To the best of your knowledge, has an event of this type in your unit—including not only treatment refusal but also the mere discussion of the issue—led to legal (lawsuit/threat of lawsuit) or disciplinary actions (such as a complaint to an external oversight body like a government ministry, hospital administration, or healthcare provider)?” In response, four directors reported legal proceedings, while two (26% in total) reported disciplinary actions. These cases are detailed in Table 2. Information on the final decisions in these cases was not collected.

**Table 2:** Cases of patients who initiated legal or disciplinary actions following referral for child welfare capacity assessment (Reported by IVF Unit Directors)

• A patient with severe mental retardation and azoospermia was under the custody of his wife. In accordance with the Israel Fertility Association guidelines, which state that a person who is not qualified to sign an informed consent is not a candidate for fertility treatments, we refused to treat. A court ruled that we must perform a surgical sperm extraction with the wife’s consent
• The unit refused to treat a patient due to the physical disability of a single mother, and a court ordered us to handle it
• A couple with psychiatric disorders refused to provide an updated psychiatric evaluation and contacted the Ministry of Health, complaining of discrimination. We received a letter demanding an explanation from the legal advisor of the Ministry of Health
• A couple was refused treatment following the recommendation of an ad hoc ethical committee (consisting of the hospital director, legal advisor, public representative, and risk management attorney), the patients’ lawyer contacted the hospital and threatened a lawsuit
• A patient with post-traumatic stress disorder and impaired hand function, treated with psychiatric medication was asked to undergo a psychiatric evaluation before continuing treatment. She was previously treated in another unit where, according to her, she was not asked for any psychiatric evaluation. After it was explained to the patient, she had to undergo evaluation before fertility treatment, she filed a complaint with the hospital administration
• We received threats of legal action, letters of complaint to the hospital management, and threats to involve the media

The survey also explored alternative measures besides referring to a CWC, such as denying treatment without CWC consultation, recommending that patients seek treatment at another unit, or deliberately prolonging assessment and the evaluation processes. Ten directors (43%) reported employing such measures, with half using them in one or two cases and the other half in more than two cases. Of these, three respondents described an internal evaluation process within their unit, and one respondent reported two cases in which a patient was denied treatment without a CWC discussion, due to refusal to undergo psychiatric follow-up for addiction to pain medications and refusal to cooperate with social welfare authorities regarding children who had been removed from her custody. Twelve directors did not describe such measures, and two did not respond to this question. Finally, respondents were asked if they would like to add comments on this topic, and eight participants provided responses. Of these, five expressed dissatisfaction with the current situation. Some emphasized the lack of support from hospital ethics committees, stating, for example:

- “In all cases we referred to the hospital ethics committee, the response was that we are required to provide treatment.”
- “There must be a solution for assessing ‘parental capacity’ before a child is born, rather than waiting until welfare authorities seek to remove them from their home.”

Two respondents described the role of the local ethics committee, while one stated that cases in which treatment is denied due to insufficient parental capacity are rare, questioning whether efforts to formalize this issue are warranted. The complete survey responses are provided in the supplementary material.

## Discussion

The issue of child welfare in the context of fertility treatments presents complex ethical dilemmas for clinicians. While the ethical dimensions of child welfare in fertility treatments are widely discussed in the literature, practical issues are seldom presented, likely due to the sensitivity of the topic. This study offers a closer look at the distribution of reasons for concern regarding patients referred by IVF directors to CWC for assessment, and directors’ experience with these bodies. The findings show that although concerns about a future child’s welfare are rare, they arise in nearly all IVF units. The most common reasons for these concerns were cognitive developmental disability, mental illness that may affect parental capacity, and severe physical disability. Notably, almost all units reported consulting a CWC at least once within the past 3 years.

Previous studies have focused on professional views through surveys and focus groups [7, 17, 22, 23], while practical issues surrounding treatment denial remain underexplored. Gurmankin et al. found that ART programs in the U.S. declined 3% of candidates for medical reasons and 1% for emotional, social, or psychological reasons [23]. A Swedish study reported a 7.6% treatment denial rate, mostly due to excess maternal weight (2.2%), while 1% were denied treatment due to psychiatric or social disorders, and a similar proportion excluded for a “physical disorder” [24]. Our study adds more detailed information on the exact reasons causing concern among IVF clinicians regarding PC.

Israel has the highest fertility rate among developed countries [25], reflecting the cultural significance of reproduction in Israeli society. The country’s generous public funding for fertility treatments removes financial barriers that often limit access to costly procedures in other countries. However, this extensive funding may also create additional pressure on fertility specialists, as it fosters a sense of obligation to provide treatment whenever it is deemed medically feasible. Some clinicians may feel compelled to offer treatment despite reservations regarding the future welfare of the child. Despite this, the legal framework surrounding treatment refusal based on future PC concerns remains unclear, leaving clinicians uncertain about their authority to deny care. This highlights the important role of CWCs as advisory bodies supporting clinicians in evaluating PC and determining whether treatment should be provided or withheld. Yet, one-third of respondents expressed dissatisfaction with their CWC’s performance or accessibility.

This dissatisfaction may reflect broader systemic ambiguity rather than committee failure, mirroring the absence of clear legal or regulatory guidance on denying treatment due to child welfare concerns. One in four respondents described instances where patients who were denied treatment or required further assessment escalated the situation by appealing, or threatening to appeal to hospital administrators, initiating legal action, or threatening media involvement. In these cases, respondents reported that CWCs or courts failed to support their decision, reflecting a sense of frustration and disappointment.

In this study, 10 of 23 respondents acknowledged using alternative management strategies beyond formal CWC discussions when concerns about PC arose. These included outright denial of IVF treatment, either without formal CWC approval or even despite committee approval. In other cases, clinicians recommended that patients seek treatment at another facility or deliberately prolonged the evaluation process to encourage them to withdraw voluntarily. Similar “delay tactics” were reported by fertility counselors in Australia [22], reflecting a common discomfort among IVF teams when directly addressing concerns about PC. These strategies may represent an attempt to avoid the ethical and legal complexities of formal treatment denial.

Taken together, these findings suggest that beyond addressing ethical and clinical dilemmas, IVF unit directors face significant external pressures and often lack sufficient professional or regulatory support when managing complex cases. This underscores the need for clear legal guidelines and well-defined decision-making frameworks, which could better support clinicians in balancing their medical, ethical, and legal responsibilities when concerns about future PC arise.

One of the unanswered questions in this area of research is the actual (not only presumed) parental competence of IVF patients who raised concerns among clinic staff prior to treatment. Addressing this question directly requires long-term follow-up of these patients to determine whether staff concerns correlate with adverse outcomes. To our knowledge, such a study has yet to be conducted. However, a study examining the characteristics of parents whose children were placed in foster care in Israel at an average age of 3.2 years identified certain parental traits. The study found that at least one parent in 83% of cases had a documented limitation (such as a diagnosed illness, disability, or cognitive disability). Additionally, in 59% of cases, at least one parent had a history of substance abuse or involvement in criminal behavior [26].

In our study, cognitive developmental disabilities, mental illness, and severe physical disability were the most common reasons for referral. Fewer than 20% of respondents cited substance abuse or criminal history as reasons for referral to CWC evaluation, likely due to these being less visible or not disclosed by patients. This raises a question debated internationally: should patients be required to declare drug or alcohol use, or even submit a police clearance, as is done in the UK and Norway [16, 17].

In this study, we surveyed IVF unit directors to examine how they address practical challenges when concerns arise about future PC. In Israel, these directors are typically personally involved in managing all controversial cases referred for evaluation by CWC within their units. Our study included 23 directors of 26 IVF units in Israel. Therefore, while the number of respondents is modest, this study provides a comprehensive perspective and near-complete national coverage on how PC concerns are managed, while avoiding the limitations of studies that survey random staff members from one or a few units.

This study is limited to IVF unit directors in Israel, which may restrict the generalizability of findings to other healthcare systems. The reliance on self-reported data introduces the possibility of recall bias.

Our study demonstrates that while concerns about future PC relatively infrequent, they are present in all IVF units. The most commonly cited concerns involve cognitive developmental disability, mental illness, and severe physical disabilities. While CWCs serve as a consultation mechanism in these cases, satisfaction with their role remains mixed, highlighting the need for clearer guidelines and improved accessibility. Further research is needed to examine the outcomes of fertility treatment and parenting among patients whose parental capacity was questioned, as well as a detailed investigation of cases referred to the CWC, including evaluation processes, committee conclusions, and the subsequent actions of IVF teams.

## Supplementary Information

Below is the link to the electronic supplementary material.

Supplementary file1 (DOCX 18 KB)

Supplementary file2 (XLSX 14 KB)

## Data Availability

Datasets are available upon request.
