# Supplementary material for: Managing parental capacity concerns in IVF: the role of child welfare committees—a survey of unit directors
Source: J Assist Reprod Genet. 2025 Jun 2;42(7):2305–11. doi: 10.1007/s10815-025-03529-y (PMC12356760; doi:10.1007/s10815-025-03529-y)
Supplement: Supplementary file 1 — Supplementary file1 (DOCX 18 KB) [file 10815_2025_3529_MOESM1_ESM.docx]

Survey for IVF Unit Directors on Fertility Treatments and Child Welfare Concerns

1. In the past three years, approximately how many times has your unit needed to discuss treatment for couples/women where concerns arose regarding the future welfare of the child?

Answer: __________(short text)

2. What were the circumstances that required such a discussion? You may select more than one answer.

- Cognitive developmental disability
- Mental illness that may affect parental capacity
- Severe physical disability
- Drug/alcohol use to an extent that may impair parental capacity
- Criminal aspects
- Other ______________

3. During this period, how many women/couples were referred, or did you wish to refer, from your unit for discussion by a 'Child Protection Committee' / institutional ethics committee, or an equivalent body?

Answer: ___________(short text)

4. In how many cases were alternative measures taken in your unit, such as staff refusal to provide treatment without a committee discussion or despite committee approval, recommending treatment at another unit, or deliberately prolonging the evaluation process until the patient/couple voluntarily withdrew?

Answer: ___________(short text)

5. What is the availability and quality of the response in your institution from the 'Child Protection Committee' / institutional ethics committee in these situations?

- Adequate response
- Response exists but is insufficient in terms of availability or quality
- No such committee exists
- We do require such a response

6. If the response is inadequate, what do you believe is the main reason for this?

- Lack of cooperation from the hospital
- No need for such a body
- Lack of legal authority for the committee’s recommendations
- Not applicable (an adequate response is available)
- Other (please specify): _______________

7. To the best of your knowledge, has an event of this type in your unit—including not only treatment refusal but also the mere discussion of the issue—led to legal (lawsuit/threat of lawsuit) or disciplinary actions (such as a complaint to an external oversight body like a government ministry, hospital administration, or healthcare provider)?

- Legal proceedings: ______ cases
- Regulatory complainד: ______ cases
- Media: _______cases
- There was no such event

8. If your answer to the previous question is positive, please provide a brief description

9. Would you like to add any comments regarding this topic?: ____________________
